# Management of BCG non-responders with fixed dose intravesical gemcitabine in superficial transitional cell carcinoma of urinary bladder

**DOI:** 10.4103/0970-1591.35759

**Published:** 2008

**Authors:** N. K. Mohanty, Rajiba L. Nayak, Pawan Vasudeva, R. P. Arora

**Affiliations:** Department of Urology, VM Medical College and Safdarjung Hospital, New Delhi, India

**Keywords:** BCG, gemcitabine, non-responders

## Abstract

**Aims and Objectives:**

The incidence of bladder malignancy is increasing worldwide and the projected rise is 28% by 2010 for both sexes (WHO). Though intravesical adjuvant therapy with BCG is superior to any other immunotherapeutic/chemotherapeutic agent in reducing tumor recurrences and disease progression, its real efficacy remains controversial as one-third of the patients will soon become BCG failure. Hence there is a need for an alternative intravesical agent for treatment of BCG failure. Our aim was to study the efficacy, tolerability and safety of intravesical Gemcitabine in managing BCG-refractory superficial bladder malignancy.

**Materials and Methods:**

Thirty-five BCG failure patients, 26 males and nine females between 20-72 years of age were instilled with 2000 mg of Gemcitabine in 50 ml of normal saline intravesically two weeks post tumor resection, for six consecutive weeks. Mean follow-up was for 18 months with cystoscopies.

**Results:**

Twenty-one patients (60%) showed no recurrences, 11 patients (31.4%) had superficial recurrences while three patients (8.6%) progressed to muscle invasiveness. Average time to first recurrence was 12 months and to disease progression was 16 months. Adverse event was low and mild. Therapy was well tolerated.

**Conclusion:**

Gemcitabine fulfils all requirements as an alternative agent, in treating BCG failure patients with low adverse events, is well tolerated and highly effective in reducing tumor recurrences.

Incidence of transitional cell carcinoma (TCC) of urinary bladder is increasing worldwide with a projected rise of 28% by 2010 for both sexes (WHO). This malignancy is particularly important for the social system of industrialized countries. After initial therapy by trans urethral resection of bladder tumor (TURBT), 75-80% will recur within one year and 20-25% will progress to muscle invasiveness if no adjuvant therapy is considered.

Intravesical instillation of Bacillus Calmitte Guerin (BCG) is the best intravesical adjuvant therapeutic agent in reducing tumor recurrences and disease progression in the intermediate-risk (Ta/T1-Gr.I-II, multifocal, size >3 cm) and high-risk (T1G3, CIS) group as per European guidelines.[1,2]

Although BCG is currently considered the most effective agent in reducing recurrence rate and disease progression rate in superficial bladder cancer (SBC), its real efficacy remains controversial. A two-year follow-up with BCG showed a 40% recurrence rate.[3] Peyromaure *et al.*,[4] reported a 42% and 28% recurrence and progression rate respectively in 57 patients followed up for 53 months with BCG therapy. Recent studies have shown that BCG is effective in only two-thirds of SBC while one-third will be non-responders.[5] Those SBC and CIS that fail to respond to initial BCG therapy are defined as BCG failures or non-responders.[6] Post BCG therapy recurrences show poor prognosis for whom radical cystectomy remains the only treatment option. The side-effect profile of 120 mg BCG represents another limiting factor for its use intravesically.

Chemotherapeutic agents such as Mitomycin-C and Doxorubicin in spite of the low probability of systemic side-effects can give rise to severe chemical cystitis.[7] Recent studies have shown good results with intravesical electromotive Mitomycin-C instillation.

Again, following conventional intravesical chemotherapy, the short-term recurrence rate of intermediate-risk SBC cannot be reduced by more than 15-20% and long-term risk of recurrence by 6% according to Lamn.[8]

Hence there is a need for a new intravesical agent for the treatment of the intermediate and high-risk SBC groups who do not respond to initial BCG therapy.

Systemic chemotherapy with gemcitabine and cisplatin for locally advanced muscle invasive bladder cancer has shown promising results[9,10] with low side-effects and good tolerance as an alternative treatment option to conventional radical cystectomy.

Gemcitabine, a deoxycytidine analogue has broad-spectrum anti-tumor activity and being a non-vesicant drug has very little local toxicity; hence high concentration in bladder could be achieved without drug-induced cystitis. It substantially penetrates bladder mucosa better, unlike Mitomycin-C, due to its lower molecular weight of 299 Da and preferentially produces cytotoxicity in bladder cancer cells while sparing non-transferred bladder mucosa and submucosal cells. Its rapid transformation into an inactive metabolite helps prevent systemic toxicity.

On the basis of the high relative advantage for regional therapy with gemcitabine and proven efficacy on systemic administration against invasive cancer bladder we undertook the following study with an aim to determine the efficacy, safety and tolerability of fixed dose intravesical gemcitabine (2000 mg) weekly for six weeks for two hours duration in only those patients with superficial bladder cancer who had experienced failure to previous intravesical therapy with BCG.

## MATERIALS AND METHODS

### Study design

This was a prospective, single-arm, non-randomized study conducted between January 2004 and December 2006.

Patients with superficial TCC of urinary bladder (Ta, T1 and all grades) who had earlier indications for BCG therapy i.e. multiple tumors, size more than 3 cm and of younger age group but had shown repeated recurrence on follow-up were recruited in our study. This is a continuous ongoing study. Other eligibility criteria to be included in this study were age more than 18 years, good performance status, normal hepatic, renal and hemocritic values and patients consent. Patients with at least one of the following criteria were excluded from this study i.e. evidence of locally invasive or metastatic bladder cancer (T2), presence of upper tract malignancy, immunotherapy or radiotherapy received within 12 weeks of initiation of the study and unresolved UTI.

Between January 2004 and December 2006, a total number 35 such BCG failure patients were included in our study with an average age of 54 years (20-72 years). All these 35 patients underwent trans urethral resection of bladder tumor. Histology confirmed superficial in nature (Ta/T1) of all grades. This subgroup showed multiple tumor recurrences of size > 3cm and designated as BCG non responders. They were subsequently subjected to intravesical instillation of gemcitabine (Gemcel) 2000 mg dissolved in 50 ml of normal saline (0.9%) weekly for six consecutive weeks. Intravesical therapy was started one week post TURBT. Each instillation lasted for 2 h. Alkalinization of urine was done during therapy by asking the patients to take systemic alkalizer a day prior to therapy so as to get better absorption of gemcitabine. Fluid intake restriction and complete emptying of bladder was done routinely prior to therapy in order to improve therapeutic efficacy. Periodic follow-up with urine for cytology, NMP22, ultrasonography and cystoscopies at three-monthly interval i.e. at third, sixth, ninth and 12^th^ month for the first year and thereafter every six-monthly was done. The total number of cystoscopies the patient underwent during 18 months follow-up was six.

## RESULTS

All these 35 patients had initially undergone intravesical therapy with BCG after TURBT since they fulfilled the inclusion criteria for adjuvant therapy with BCG i.e. Ta/T1 stage, multiple tumors and size more than 3 cm. On periodic follow-up they showed repeated multiple recurrences hence were designated as BCG refractory which entitled them to receive intravesical gemcitabine. The histology grade of these 35 patients before therapy is shown in Table 1.

**Table 1:** Pre-therapy histology of patients

Stage	No. of patients	Grade	No. of pts.
Ta	18 (M-13, F-5)	I	5
		II	8
		III	5
T1	17 (M-13, F-4)	I	3
		II	6
		III	8

At the end of 18 months follow-up 21 patients (60%) showed no tumor recurrences, 11 patients (31.4%) showed superficial tumor recurrences and three patients (8.6%) showed muscle invasiveness. Average time to first recurrence was 12 months and average time to disease progression was 16 months.

Four patients (8.75%) had side-effects in the form of nausea, cystitis, frequency of urination, hematuria and neutropenia [Table 2]. None of these 35 patients discontinued therapy due to adverse effects. All instillation was for 2 h weekly for six consecutive weeks. Patients' compliance was good.

**Table 2:** Adverse effects - 4/35 (8.75%)

Symptoms	No. of patients	% age
Nausea	4	8.75
Cystitis	4	8.75
Frequency	4	8.75
Hematuria	1	2.86
Neutropenia Gl	1	2.86

In our series [Table 1] a total number of eight patients having Grade I tumor (Ta-5 and T1-3) had multiple tumors (more than three and size >3cm) at the beginning hence it was thought appropriate to start intravesical adjuvant BCG on them initially after TURBT as they were falling under the intermediate risk group as per European Guidelines.

## DISCUSSION

Pharmacokinetic data from different phase studies clearly demonstrates that systemic absorption of intravesical gemcitabine is minimal and transient and this is unlikely to produce clinically significant adverse events.

*In vitro* cytotoxic effects of gemcitabine in intravesical therapy showed selective cytotoxicity to human and rodent TCC cell line with relative sparing of fibroblast suggesting its effectiveness in SBC.[11] Another *in vitro* study comparing gemcitabine, epirubicin, mitomycin-C and adriamycin on TCC cell culture showed cytotoxic activity 90% lethality to TCC with gemcitabine as compared to the other drugs mentioned for which it was less than 60%.[12]

Recent studies using the marker lesions concept to measure the ablative efficacy of intravesical gemcitabine on intermediate-risk SBC have shown 56% complete response[13] and 50% complete response in high-risk SBC.[14]

Efficacy of intravesical gemcitabine on BCG failure cases has shown encouraging results.[15] Bouzid K *et al.*,[16] showed reduction in tumor recurrences in BCG refractory cases to be very significant with one-year follow-up. Similar positive results were published by Berardinist De *et al.*,[17] Gontero P *et al.*,[13] Dalbagni G *et al.*[14] and Serretta V.[18]

Laufer[19] in his study has shown that plasma concentration of gemcitabine decreased during the time that gemcitabine was left in the bladder indicating that it is the initial influx of gemcitabine into the bladder rather than the presence of the drug in the bladder which may be critical for systemic absorption.

Based on these experimental findings and favorable pharmacokinetic properties of gemcitabine, the role of its intravesical use on BCG refractory SBC patients seems to be not only feasible but also scientific with a good response rate.

Our study of 35 BCG failure patients with fixed dose of intravesical gemcitabine (2 g) weekly for 2 h for six consecutive weeks with 18 months follow-up showed very encouraging results and in 91.4% of these patients the bladder could be salvaged for 18 months, otherwise cystectomy would have to be performed.

Intravesical gemcitabine has so far shown an excellent safety profile with minimal toxicity at a concentration of 40 mg/ml.[19] Instillation time of one and two hours have both been tested with excellent tolerability.[13] Based on this excellent safety profile of intravesical instillation, as early as 3 h post TURBT has been recently advocated. Because of its low and transient adverse events and excellent cytotoxic effect, more intense treatment schedules are now being contemplated like every four weeks after initial dose of weekly for six weeks or six-instillation course twice a week for three weeks or even a 12-instillation course biweekly for six weeks.

Though our study is small it is very encouraging. However, a larger randomized clinical trial in future is needed to authenticate our results.

## CONCLUSION

Intravesical gemcitabine seems to have fulfilled all the requirements of an alternative agent in managing BCG failure patients with superficial bladder cancer in whom bladder can be salvaged but warrants further investigation with respect to its anti-tumor activity with marker lesion concept, its tolerability and long-term efficacy.
